# The Threat of Antibiotics and Heavy Metal Pollution to Human Health and Potential Solutions

**DOI:** 10.1155/2020/8730406

**Published:** 2020-05-05

**Authors:** Peng Bao, Xiu-Li Gao, Guo-Qi Wen

**Affiliations:** ^1^Key Lab of Urban Environment and Health, Institute of Urban Environment, Chinese Academy of Sciences, Xiamen 361021, China; ^2^Guizhou Medical University, Guiyang, China; ^3^Université Laval, Québec, Canada

Environment-associated human health problems are getting worldwide attention with the extension of human society. Antibiotics are abused to protect the health of humans and animals that may accelerate the development of antibiotic resistance genes (ARGs) and antibiotic-resistant microorganisms (ARMs) which shade health risks to humans. A wide variety of ARGs have been found in microorganisms distributed in the environment. Heavy metal contamination represents a long-standing selection pressure on the maintenance and spread of ARGs, with both environmental and clinical importance. Therefore, heavy metals have also led to their own concerns over their risks on human health and the environment. In this special issue, we intend to report research studies that focus on adverse effects of antibiotics and heavy metals on the environment and human health and meta-analysis, medicines, and technologies that improve human health. The purpose of this special issue is to report the recent progress of those fields. A brief summary of all accepted papers is provided below.

The paper by Wang et al. reported that metformin significantly improved high blood pressure, proteinuria, and the foetal and placental weights in the HF-M group compared with the HF group. Metformin significantly improved placental labyrinth and foetal vascular development in preeclampsia. In addition, metformin effectively increased matrix metalloproteinase-2 (MMP-2) and vascular endothelial growth factor (VEGF) levels in the placenta.

The paper by Zhang et al. reported that HER2+BC patients showed improvement in pCR and DFS after neoadjuvant trastuzumab treatment. Patients without pCR had prolonged DFS after trastuzumab maintenance.

The paper by Asha et al. reported an increased abundance of antibiotic resistance genes in the kidney transplant subjects. The antibiotic resistance genes identified in the transplant subjects were predominantly composed of multidrug efflux pumps (MDEPs) which are evolutionarily ancient, are commonly encoded on chromosomes rather than plasmids, and have a low rate of mutation. Since the MDEPs had a low abundance in the healthy subjects, the MDEPs may enhance the fitness of bacteria to survive in the high-stress environment of transplantation that includes multiple stressors including surgery, antibiotics, and immunosuppressive agents.

The paper by Hao et al. found that cardiac contractility modulation (CCM) could elicit positive effects on heart failure (HF) therapy, which was potentially due to the variation in the Pi3k/Akt signaling pathway.

The paper by Guo et al. reported a heavy metal removal process that was optimised by orthogonal experiment with dynamic and static adsorption modes. The method can be used to satisfy the high efficiency of selective removal of harmful elements in Acanthopanax senticosus extract and the effective composition of almost no effect, it can be recommended for pretreatment of heavy metals in traditional Chinese medicine extracts, and this way provides a new thought and research technique to decrease the contents of heavy metals.

The paper by Su et al. found that LESS surgery with conventional laparoscopic instruments is feasible and safe compared with traditional laparoscopy, but postoperative exhaust time is longer than the control group.

The paper by Fang et al. reported that the quality control circle activities played a significant role in promoting the use of rubber dams in the root canal treatment of primary teeth, and it can be used as a method to promote new clinical treatment programs.

The paper by Chen et al. reported that free flap transfer is an ideal method for the reconstruction of large, complicated scalp defects with a one-stage operation. The anterolateral thigh flap is favored because of its durability, adjustability, water tightness, and infection prevention.

